# Complex antigen presentation pathway for an HLA-A*0201-restricted epitope from Chikungunya 6K protein

**DOI:** 10.1371/journal.pntd.0006036

**Published:** 2017-10-30

**Authors:** Elena Lorente, Alejandro Barriga, Juan García-Arriaza, François A. Lemonnier, Mariano Esteban, Daniel López

**Affiliations:** 1 Unidad de Procesamiento Antigénico, Centro Nacional de Microbiología, Instituto de Salud Carlos III, Majadahonda, Madrid, Spain; 2 Department of Molecular and Cellular Biology, Centro Nacional de Biotecnología, Consejo Superior de Investigaciones Científicas (CSIC), Madrid, Spain; 3 Unité d'Immunité Cellulaire Antivirale, Département d'Immunologie, Institut Pasteur, France; University of Glasgow, UNITED KINGDOM

## Abstract

**Background:**

The adaptive cytotoxic T lymphocyte (CTL)-mediated immune response is critical for clearance of many viral infections. These CTL recognize naturally processed short viral antigenic peptides bound to human leukocyte antigen (HLA) class I molecules on the surface of infected cells. This specific recognition allows the killing of virus-infected cells. The T cell immune T cell response to Chikungunya virus (CHIKV), a mosquito-borne *Alphavirus* of the *Togaviridae* family responsible for severe musculoskeletal disorders, has not been fully defined; nonetheless, the importance of HLA class I-restricted immune response in this virus has been hypothesized.

**Methodology/Principal findings:**

By infection of HLA-A*0201-transgenic mice with a recombinant vaccinia virus that encodes the CHIKV structural polyprotein (rVACV-CHIKV), we identified the first human T cell epitopes from CHIKV. These three novel 6K transmembrane protein-derived epitopes are presented by the common HLA class I molecule, HLA-A*0201. One of these epitopes is processed and presented via a complex pathway that involves proteases from different subcellular locations. Specific chemical inhibitors blocked these events in rVACV-CHIKV-infected cells.

**Conclusions/Significance:**

Our data have implications not only for the identification of novel *Alphavirus* and *Togaviridae* antiviral CTL responses, but also for analyzing presentation of antigen from viruses of different families and orders that use host proteinases to generate their mature envelope proteins.

## Author summary

The arboviral pathogen Chikungunya virus (CHIKV) is a serious threat to global health, and is considered a priority re-emerging virus. This pathogen causes acute febrile infection in patients, leading to debilitating arthralgia and arthritis. In recent years, CHIKV has spread quickly in tropical and subtropical countries, causing outbreaks of more severe forms of the disease than previously reported. The nature and function of the T cell immune response, critical for clearance of viral infections, is largely unknown during acute and chronic CHIKV disease and their association with rheumatic disorders. In this study, we identified the three first CHIKV epitopes recognized by human T cells. We studied how one of these epitopes is generated in virus-infected cells, a process that involves the sequential proteolytic activity of several proteases at distinct subcellular locations. We postulate that this process could have broad implications when applied to other viral proteins.

## Introduction

The mosquito-borne Chikungunya virus (CHIKV), a member of the *Alphavirus* genus of the *Togaviridae* family, causes an acute febrile infection in patients that leads to debilitating arthralgia and arthritis. Identified in the former Tanganyika territory in 1952 [1–3], this arboviral pathogen caused numerous epidemics in Africa and Asia from the 1960s–1980s [4, 5]. Following several decades of relative inactivity, CHIKV re-emerged in 2005 to cause an explosive epidemic in the Indian Ocean area, mainly on Reunion Island. In this French overseas department, the outbreak affected about half of its 700,000 inhabitants, with more than 250 deaths [5]. In 2006, several million people were infected by this virus in another large outbreak in India [6]. In recent years, this infectious disease has spread quickly from Africa and Asia to the Americas [7], causing outbreaks in tropical and subtropical countries of more severe forms than previously reported [8,9]. Morbidity due to CHIKV infection is a serious threat to global health and this virus is considered a priority emerging pathogen [10].

CHIKV is an enveloped virus with a positive-sense, single-stranded RNA genome that encodes two large polyproteins [11]. The nonstructural P1234 precursor is autocatalytically processed by the C-terminal domain of the nonstructural protein 2 (nsP2) and releases the four multifunctional nsP proteins. In contrast, in maturation of the structural polyprotein, viral and host proteases are both involved in producing capsid, E1, E2, and E3 envelope and 6K transmembrane proteins [11].

Although the immune mechanisms involved in CHIKV disease are not fully understood, CHIKV-infected humans show CD8^+^ T lymphocyte responses in early disease stages [12]; a large percentage of these activated CD8^+^ T cells can be detected more than 7 weeks postinfection in patient blood samples [13]. The nature and function of CD8^+^ T cells during acute and chronic CHIKV infection is largely unknown, as is their association with rheumatic disorders. Although the importance of the HLA class I-restricted immune response has been hypothesized [14], to date, no human T cell epitope has been described in CHIKV infection.

In cellular immunity, CD8^+^ T lymphocytes recognize short viral peptides exposed at the membrane of infected cells [15]. Most of these epitopes are generated by proteolytic degradation of the fraction of newly synthesized viral proteins whose sequence or folding are in some way defective (defective ribosomal products; DRiP) and are thus degraded immediately by the combined action of proteasomes and other cytosol degradative peptidases [16]. The antigen processing products are translocated to the endoplasmic reticulum (ER) lumen by transporters associated with antigen processing (TAP), where N-terminal trimming by the ER aminopeptidase (ERAP) is frequently necessary [17,18]. Some of these final peptides might bind the human histocompatibility complex (human leukocyte antigen; HLA) class I heavy chain and β_2_-microglobulin. The stable trimolecular peptide-HLA-β_2_-microglobulin complexes are then exported to the cell surface for cytotoxic T lymphocyte (CTL) recognition [15]. In addition to this classical antigen processing pathway, several alternative routes have been described that contribute to endogenous HLA class I-restricted antigen processing (reviewed in [19]). During maturation of the viral structural polyprotein, the short CHIKV 6K transmembrane protein is efficiently cleaved by the host ER signal peptidase, rendering it a possible source of viral epitopes via alternative pathways. To search for CHIKV 6K protein T cell epitopes, we infected HLA-A*0201-transgenic mice with a recombinant vaccinia virus that encodes the CHIKV structural polyprotein; we identified three epitopes presented by the HLA class I molecule, one of which is processed and presented in a pathway that involves proteases from distinct subcellular locations.

## Materials and methods

### Ethics statement

H-2 class I knockout HLA-A*0201-transgenic mice [20], a versatile animal model for the study of viral and cancer antigen processing and presentation by the human major histocompatibility complex, were bred in the animal facilities at Centro Nacional de Microbiología, Instituto de Salud Carlos III, in strict accordance with the recommendations in the Guide for the Care and Use of Laboratory Animals of the Spanish Comisión Nacional de Bioseguridad of the Ministerio de Medio Ambiente y Medio Rural y Marino (accreditation n° 28079-34A). The protocol was approved by the Research Ethics and Animal Welfare Committee of the Carlos III Health Institute (permit n°: PI-283). All surgery was performed under isoflurane anesthesia, and all efforts were made to minimize suffering.

### Cell lines

The murine cell line RMA-S (TAP negative) transfected with HLA-A*0201 α1α2 domains, and the mouse H-2D^b^ α3 transmembrane and cytoplasmic domains have been described [21]. The cell line was cultured in RPMI 1640 medium supplemented with 10% fetal bovine serum (FBS) and 5 μM β-mercaptoethanol (β-ME).

### Construction of recombinant VACV-CHIKV (rVACV-CHIKV)

A vaccinia virus (VACV) Western Reserve (WR) strain expressing the CHIKV structural genes (rVACV-CHIKV) was constructed by inserting the capsid (CP), E3, E2, 6K and E1 structural genes of CHIKV clone LR2006-OPY1 into the TK locus of the WR genome [22]. The rVACV-CHIKV virus expresses the same CHIKV structural genes as those in the reported MVA-CHIKV vaccine candidate [22]. The WR strain used as the parental vector to generate rVACV-CHIKV is an optimized attenuated WR with deletions in the vaccinia immunomodulatory genes A48R, B19R and C11R (manuscript in preparation). CHIKV structural gene expression is under the transcriptional control of the viral synthetic early/late promoter. The rVACV-CHIKV virus was generated, grown in primary chicken embryo fibroblast cells and purified through two 36% (w/v) sucrose cushions. Correct CHIKV gene insertion was confirmed by PCR and sequencing, and correct CHIKV protein expression was analyzed by western blot. rVACV-CHIKV was free of contamination with mycoplasma, bacteria or fungi.

### Synthetic peptides

Peptides were purchased from Biomatik (Cambridge, Ontario, Canada). The correct molecular mass and composition of the peptides at >90% purity was established by quadrupole ion trap micro-high performance liquid chromatography (HPLC).

### Inhibitors

Brefeldin A (BFA) and all protease inhibitors were purchased from Sigma-Aldrich (Saint Louis, MO, USA), with the exception of lactacystin (from Dr. E.J. Corey, Harvard University, Cambridge, MA, USA), leupeptin (Amersham, Little Chalfont, Bucks., UK), pepstatin (Boehringer Mannheim, Mannheim, Germany), and Z-VAD-FMK (Enzyme System Products, Livermore, CA, USA). The specificity of inhibitors used is *summarized in* Table 1.

**Table 1:** General specificity of inhibitors used in this study.

Inhibitor ^a^	Abbreviation	Specificity	Reference	Concentration
Lactacystin	LC	Proteasome chymotryptic and tryptic activities	[38,39]	10 μM
Chloroquine	CQ	Lysosomotropic agent	[60,61]	50 μM
Brefeldin	BFA	Vesicle transport	[31,32]	5 μg/ml
1–10 Phenanthroline	PHE	All metalloproteases and caspase-1	[35,62]	50 μM
Leupeptin	LEU	Trypsin-like proteases and cysteine proteases	[34]	100 μM
Puromycin	PURO	Cytosol alanyl aminopeptidase and lysosomal dipeptidyl-peptidase II	[63]	0.5 μg/ml
Pepstatin	PEPST	Aspartic proteases	[34,35]	100 μM
E64	E64	Cysteine proteases C1	[33]	100 μM
Leucinthiol	Leu-SH	Metallo-aminopeptidases including ERAP	[40]	30 μM
Diazoacetyl-D,L-norleucine methyl ester	DANLME	Aspartic proteases A1	[64]	100 μM
Decanoyl-Arg-Val-Lys-Arg-chloromethylketone	dec-RVKR	Furin and other proprotein convertases	[65]	100 μM
1,3-di-(N-benzyloxycarbonyl-Leu-Leu)amino acetone	Z-LL_2_	Signal peptide peptidase	[66,67]	100 μM
Ethylenediaminetetraacetic acid	EDTA	Most metallopeptidases and some cysteine proteases	[68]	100 μM
Phenylmethane sulfonylfluoride	PMSF	Serine peptidases	[36]	1mM
Soybean Kunitz trypsin inhibitor	SBTI	Trypsin and to a lesser extent chymotrypsin and plasmin	[69]	100 μg/ml
Bestatin	BEST	Most of metallo-aminopeptidases	[35]	50 μM
Carbobenzoxy-Val-Ala-Asp-[O-methyl]- fluoromethylketone	Z-VAD	Caspases	[70]	100 μM

^a^ All inhibitors, except Z-VAD that block apoptosis, prevent proteolytic degradation in cellular extracts as measured in reference [26].

### HLA ligand prediction

SYFPEITHI software (http://www.syfpeithi.de/Scripts/MHCServer.dll/EpitopePrediction.htm) was used to predict HLA-A*0201-specific ligands of the 61-residue CHIKV 6K protein.

### HLA/peptide stability assays

Two synthetic peptides were used as positive and negative controls in complex stability assays, VACV A10L_688-696_ (ILDRIITNA, HLA-A*0201-restricted) [23] and CMV pp65_7-15_ (RCPEMISVL, HLA-C*01-restricted) [24], respectively. HLA-A*0201 RMA-S transfectants were incubated in RPMI 1640 medium with 10% heat-inactivated FBS (16 h, 26°C). Cells were washed and incubated in the same medium (2 h, 26°C) with different peptide concentrations, further incubated (2 h, 37°C), and collected for flow cytometry. HLA levels were measured using the PA2.1 monoclonal antibody (anti-HLA-A*02; Abnova, Taipei, Taiwan), as described [25]. Samples were acquired on a FACSCanto flow cytometer (BD Biosciences, San Jose, CA, USA) and analyzed with FlowJo software (TreeStar Inc, Ashland, OR, USA). The fluorescence index (FI) was calculated as the ratio of the mean channel fluorescence of the sample to that of control cells incubated without peptides. Peptide binding was also expressed as the EC_50_, which is the molar concentration of peptides that produces 50% maximum fluorescence in a concentration range between 0.001 and 100 μM.

### Generation of CD8^+^ T cell lines

Polyclonal CHIKV 6K peptide-monospecific CD8^+^ T cell lines were generated by immunizing transgenic mice with 10^7^ plaque-forming units (PFU) of rVACV-CHIKV [26]. Splenocytes from immunized mice were restimulated *in vitro* with mitomycin C-treated spleen cells pulsed with 10^−6^ M peptide and cultured Minimum Essential Medium (Alpha modification; α-MEM) with 10% FBS, 10^−7^ M peptide and 5 μM β-ME. Recombinant human interleukin-2 used for long-term propagation of peptide-specific CD8^+^ T cell lines was generously provided by Hoffmann-LaRoche (Basel, Switzerland).

### Bone marrow-derived dendritic cells

Freshly prepared bone marrow cells were cultured in 200 U/ml GM-CSF (granulocyte-macrophage colony-stimulating factor; PeproTech, London, UK), which was renewed on days 3 and 6. After 7 days, nonadherent cells with a typical dendritic cell (DC) morphology and a myeloid DC phenotype (MHC class II^+^, CD11c^+^, CD8^−^) were collected as described [27].

### IFNγ-secreting cell detection by intracellular cytokine staining (*ICS*)

ICS assays to detect recognition of peptide-pulsed or infected DC from HLA-A*0201-transgenic mice by polyclonal CTL cell lines were performed as reported [28]. Briefly, CD8^+^ T cell lines were stimulated (4 h) in the presence of 5 μg/ml BFA and of target DC previously infected with VACV-WR strain or rVACV-CHIKV (16 h). Cells were then incubated with FITC-conjugated anti-CD8 monoclonal antibody (mAb; ProImmune, Oxford, UK; 30 min, 4°C), fixed with Intrastain kit reagent A (DakoCytomation, Glostrup, Denmark), and incubated with phycoerythrin (PE)-conjugated anti-interferon (IFN)γ mAb (BD PharMingen, San Diego, CA, USA) in Intrastain kit permeabilizing reagent B (30 min, 4°C). Events were acquired and analyzed as for MHC/peptide stability assays.

When protease inhibitors were used, all drugs were added 15 min before the virus and maintained at a 2-fold higher concentration during the 1-h adsorption period than during infection. After washing the virus inoculum, inhibitors were maintained at indicated concentrations for individual experiments. The inhibitors were not toxic at these concentrations, as they did not affect antigen presentation by the VACV D12I_251-259_-specific CD8^+^ T cell line.

### Statistical analysis

To analyze statistical significance, an unpaired Student *t* test was used. *P* values <0.05 were considered significant.

## Results

### Selection of potential candidate HLA-A*0201 epitopes from CHIKV 6K protein

**Fig 1:**
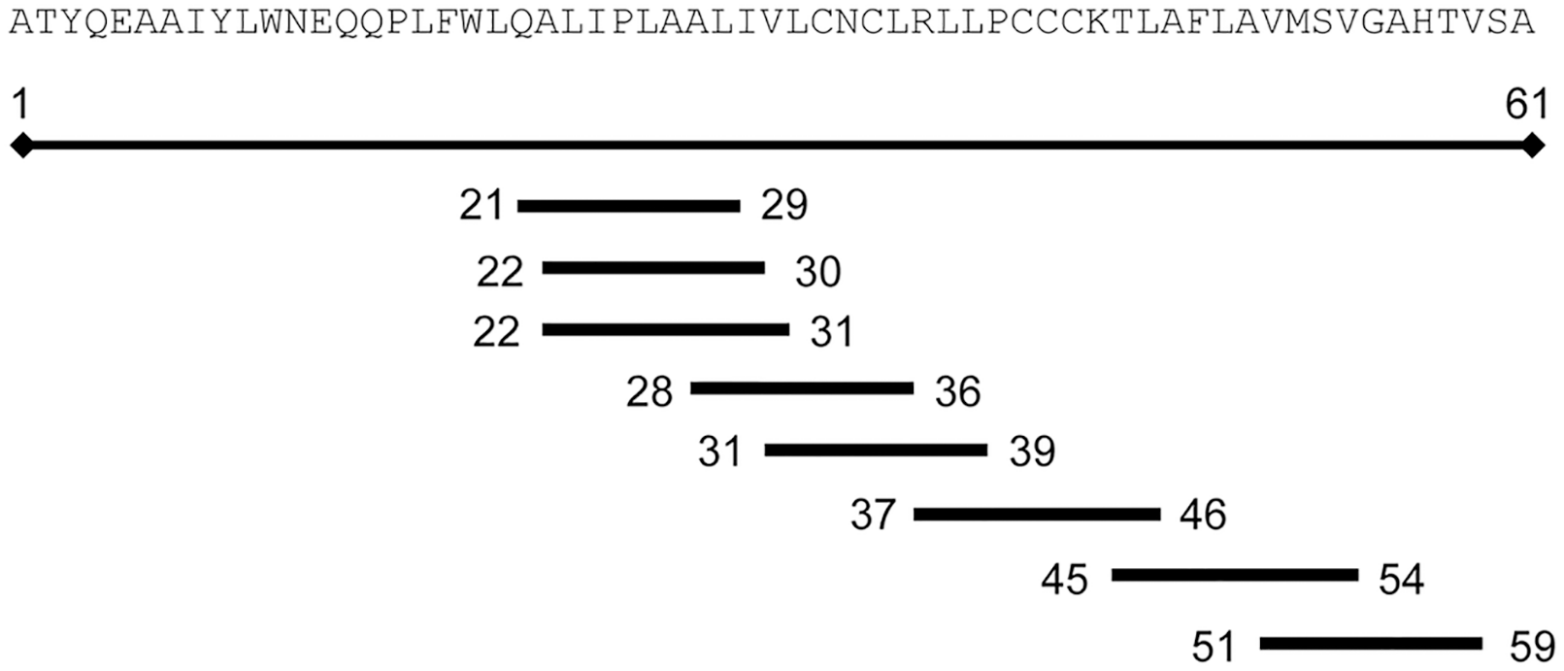
Location of potential HLA-A*0201-restricted ligands from CHIKV 6K protein. Sequence and scheme of CHIKV 6K protein, with the position of eight potential HLA-A*0201 ligands predicted by the SYFPEITHI software.

The epitope prediction tool SYFPEITHI, a reverse immunology algorithm for MHC ligand motifs [29], was used to identify possible candidate HLA-A*0201-binding peptides from CHIKV 6K protein. The five nonamers and three decamers ranked as potential HLA-A*0201 ligands (score >20) are depicted in Fig 1.

**Fig 2:**
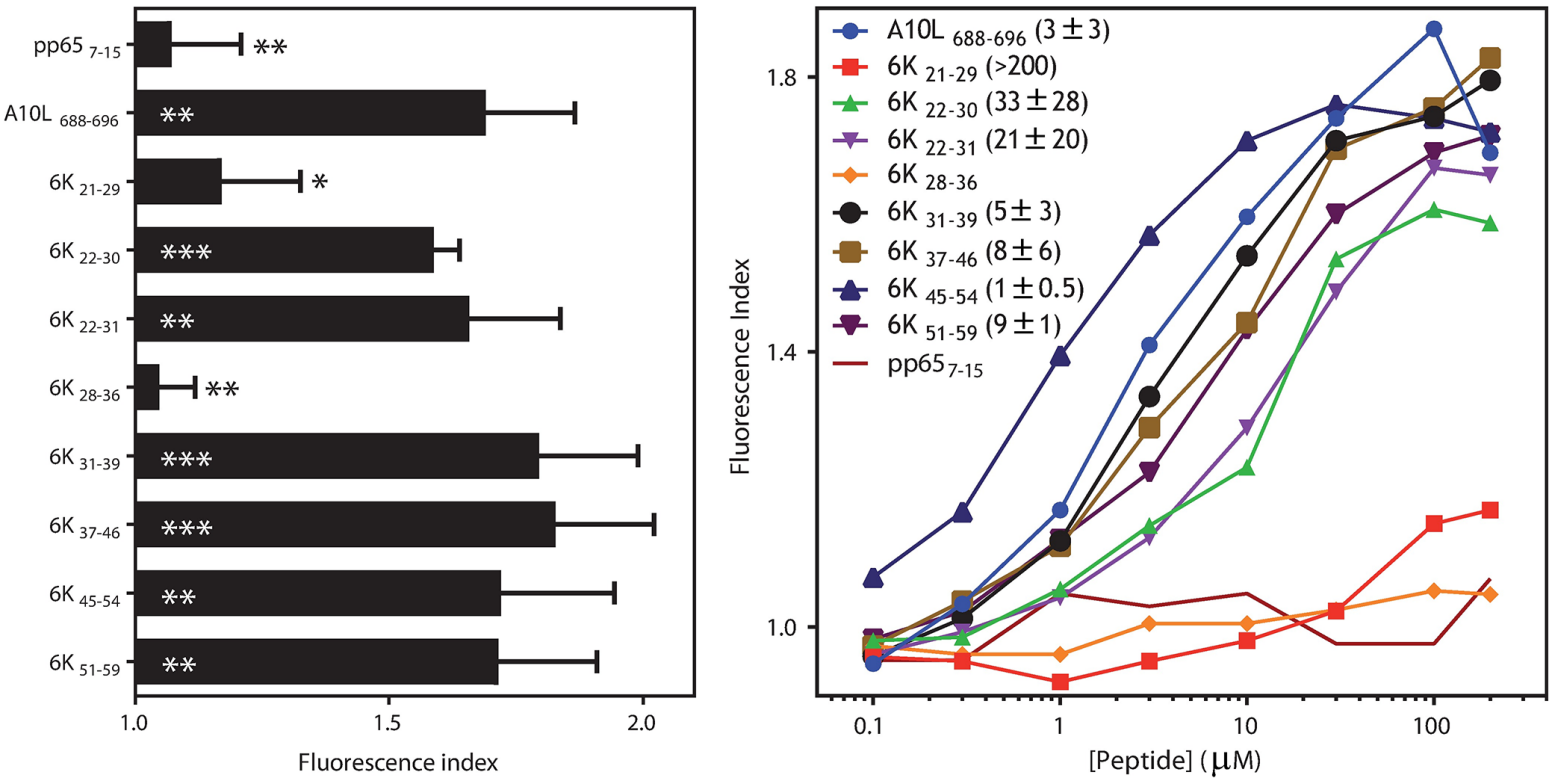
HLA-A*0201 stabilization with CHIKV 6K synthetic peptides. The stability of HLA-A*0201-peptide complexes on the surface of transfected RMA-S cells was measured by flow cytometry. Left, the indicated peptides were used at 200 μM. The CMV pp65 and VACV A10L peptides were used as negative and positive controls, respectively. The mAb PA2.1 was used for staining. The results, calculated as fluorescence indexes, are shown as the mean ± SD of four independent experiments (*** p <0.001; ** p <0.01; * p <0.05 vs. negative or positive peptide controls are represented as white or black asterisks, respectively). Right, titration curves for the indicated synthetic peptides with HLA-A*0201. Results are shown as mean values from four independent experiments. Calculated EC_50_ values (μM; mean ± SD) are shown in the right panel insert.

To study the binding ability of the eight predicted peptides to the HLA-A*0201 molecule, we performed MHC-peptide complex stability assays using HLA-A*0201-transfected, TAP-deficient RMA-S cells. Four peptides (6K_31-39_, 6K_37-46_, 6K_45-54_, 6K_51-59_) were bound to the HLA-A*02:01 class I molecules (Fig 2), with EC_50_ values in the range commonly found among natural high-affinity ligands such as the VACV A10L HLA-A*0201 epitope. In contrast, HLA affinity was substantially lower for 6K_22-30_ and 6K_22-31_ peptides, and both were considered medium-affinity ligands (Fig 2). 6K_21-29_ peptide binding to HLA-A*02:01 was residual, with a EC_50_ value >200 **μ**M (Fig 2). Stable numbers of HLA-peptide surface complexes were not detected with the 6K_28-36_ peptide (Fig 2). These data suggest that most of these peptides could be presented by the HLA-A*02:01 molecule in CHIKV-infected cells.

### Identification of three CHIKV 6K-derived HLA-A*0201 epitopes

In contrast to HLA-B*0702 transgenic mice, in which strong *ex vivo* VACV-specific T cell responses were detected [28], peptide-specific IFNγ-secreting cells from VACV-immunized HLA-A*0201 transgenic mice were usually detected only after *in vitro* stimulation. The cause of these differences is unclear, especially as both transgenic mouse types were generated in the same laboratory [20,30].

**Fig 3:**
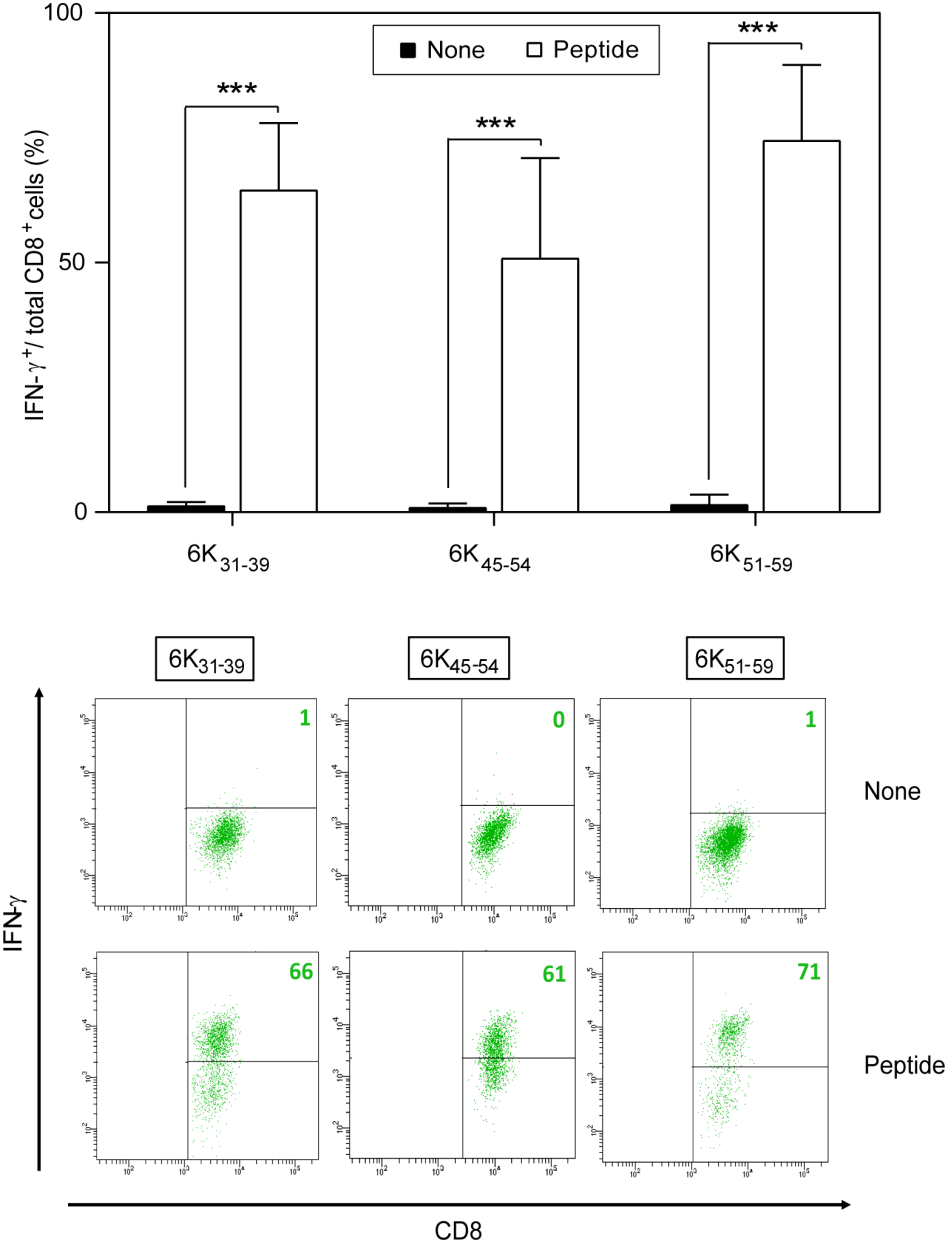
CHIKV 6K peptide specificity of HLA-A*0201-restricted CD8^+^ T cell lines. Mouse HLA-A*0201^+^ DC pre-pulsed with 10^−6^ M of indicated CHIKV 6K synthetic peptide were analyzed by ICS for CD8^+^ T cell activation with CHIKV peptide-specific CD8^+^ T cells from HLA-A*0201-transgenic mice immunized with rVACV-CHIKV and restimulated *in vitro* with the appropriate CHIKV 6K synthetic peptide. Graph data shown as mean ± SD of four independent experiments (*** P <0.001). Representative ICS panels with non-specific or CHIKV peptide-specific CD8^+^ T cell lines are depicted beneath the graphs. The percentages of IFNγ-expressing CD8^+^ T cells are indicated in each dot plot.

From rVACV-CHIKV-immunized HLA-A*0201 transgenic mice, we produced polyclonal CTL lines monospecific for each of seven CHIKV 6K peptides with stable numbers of HLA-peptide surface complexes detected in MHC-peptide complex stability assays (Table 2). The CTL lines stimulated with three of the four HLA-A*0201 high-affinity peptides (6K_31-39_, 6K_45-54_, 6K_51-59_) specifically recognized peptide-pulsed DC (Fig 3). There was no specific recognition of peptide-pulsed cells by the other four CHIKV 6K peptides (6K_21-29_, 6K_22-30_, 6K_22-31_, and 6K_37-46_; Table 2); this lack of response was confirmed using several immunization and *in vitro* stimulation protocols (not shown).

**Table 2:** Summary of CHIKV 6K peptide recognition by specific CD8^+^ T cell lines.

CHIKV peptide	Sequence	HLA-A*0201 Affinity values ^a^	CTL Line ^b^
**6K _21–29_**	QALIPLAAL	> 200	-
**6K _22–30_**	ALIPLAALI	33 ± 28	-
**6K _22–31_**	ALIPLAALIV	21 ± 20	-
**6K _28–36_**	ALIVLCNCL	No binding	N.D.
**6K _31–39_**	VLCNCLRLL	5 ± 3	+
**6K _37–46_**	RLLPCCCKTL	8 ± 6	-
**6K _45–54_**	TLAFLAVMSV	1 ± 0.5	+
**6K _51–59_**	VMSVGAHTV	9 ± 1	+

^a^ Data are expressed as EC_50_ (μM) ± SD

^b^ Measured as IFNγ secretion using ICS assays;

N.D., not done

These data indicated that CHIKV 6K_31-39_, 6K_45-54_, and 6K_51-59_ peptides are HLA-A*0201-restricted CTL epitopes, and were recognized simultaneously as part of the memory response to rVACV-CHIKV. The small (61-residue) CHIKV 6K protein thus contains at least three distinct HLA-A*0201-restricted epitopes, two of which overlap partially.

### Endogenous processing of the CHIKV 6K_51-59_ epitope

**Fig 4:**
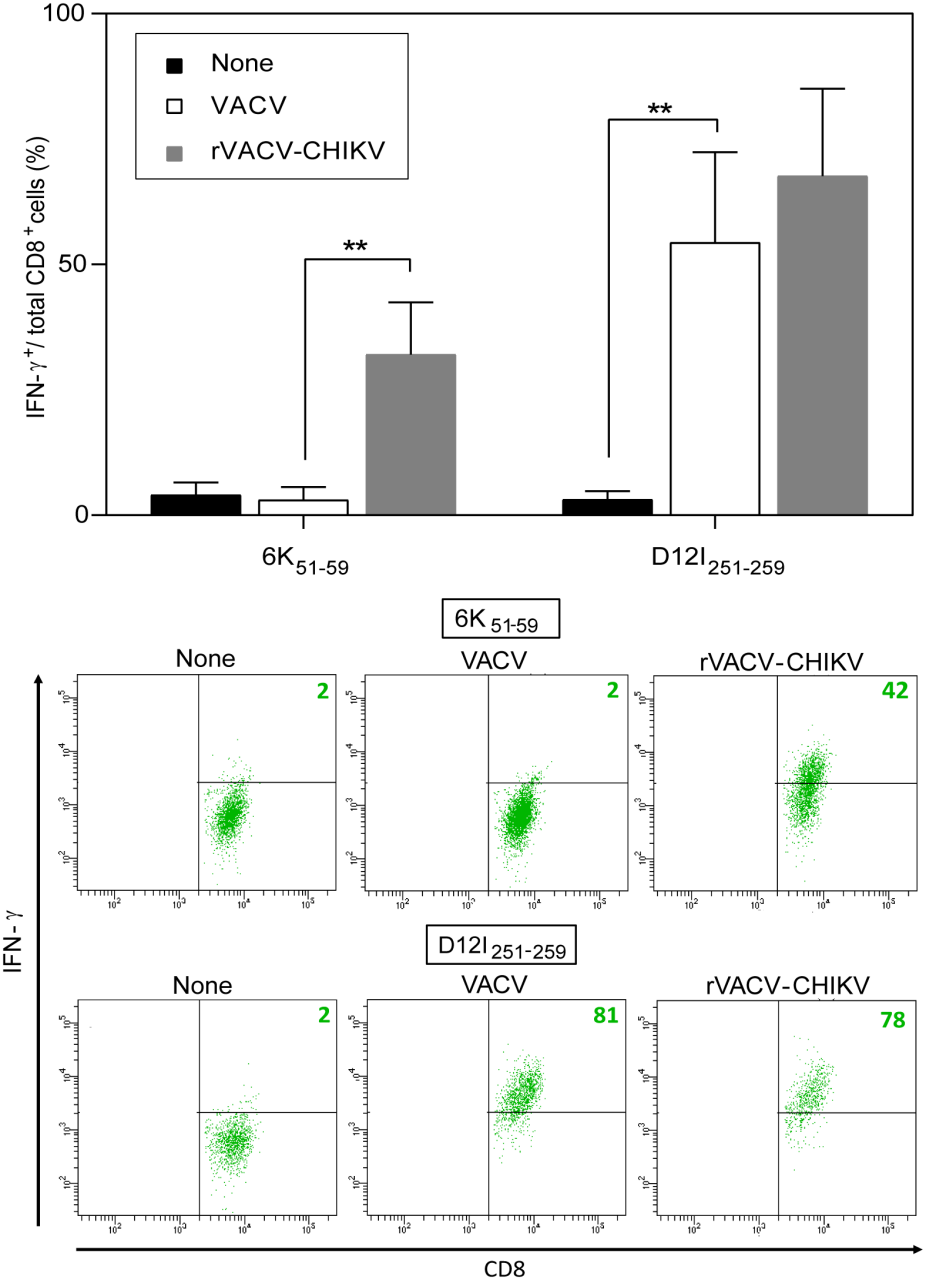
Recognition of infected HLA-A*0201^+^ dendritic cells by CHIKV 6K_51-59_- or VACV D12I_251-259_-specific CD8^+^ T cells. Mouse HLA-A*0201^+^ DC infected with VACV or rVACV-CHIKV (m.o.i. 10 pfu/cell; 5 h) were used in an ICS assay to test for recognition by CHIKV 6K_**51-59**_- or VACV D12I_251-259_-specific CD8^+^ T cell lines. Graph data shown as mean ± SD of four independent experiments (** P <0.01). Below the graphs, representative ICS panels for CHIKV 6K_**51-59**_- or VACV D12I_251-259_-specific CD8^+^ T cell lines are shown. The percentages of IFNγ-expressing CD8^+^ T cells are indicated in each dot plot.

As the three CHIKV 6K viral epitopes derive from the same 6K protein, we studied the CD8^+^ CTL line specific for the CHIKV 6K_51-59_ epitope as a representative of antigen processing of this viral protein. The CHIKV 6K_51-59_ epitope-specific CD8^+^ CTL line specifically recognized rVACV-CHIKV- but not wild type VACV-infected cells, while another T cell line specific for VACV D12I peptide 251–259 recognized both infected cells (Fig 4).

**Fig 5:**
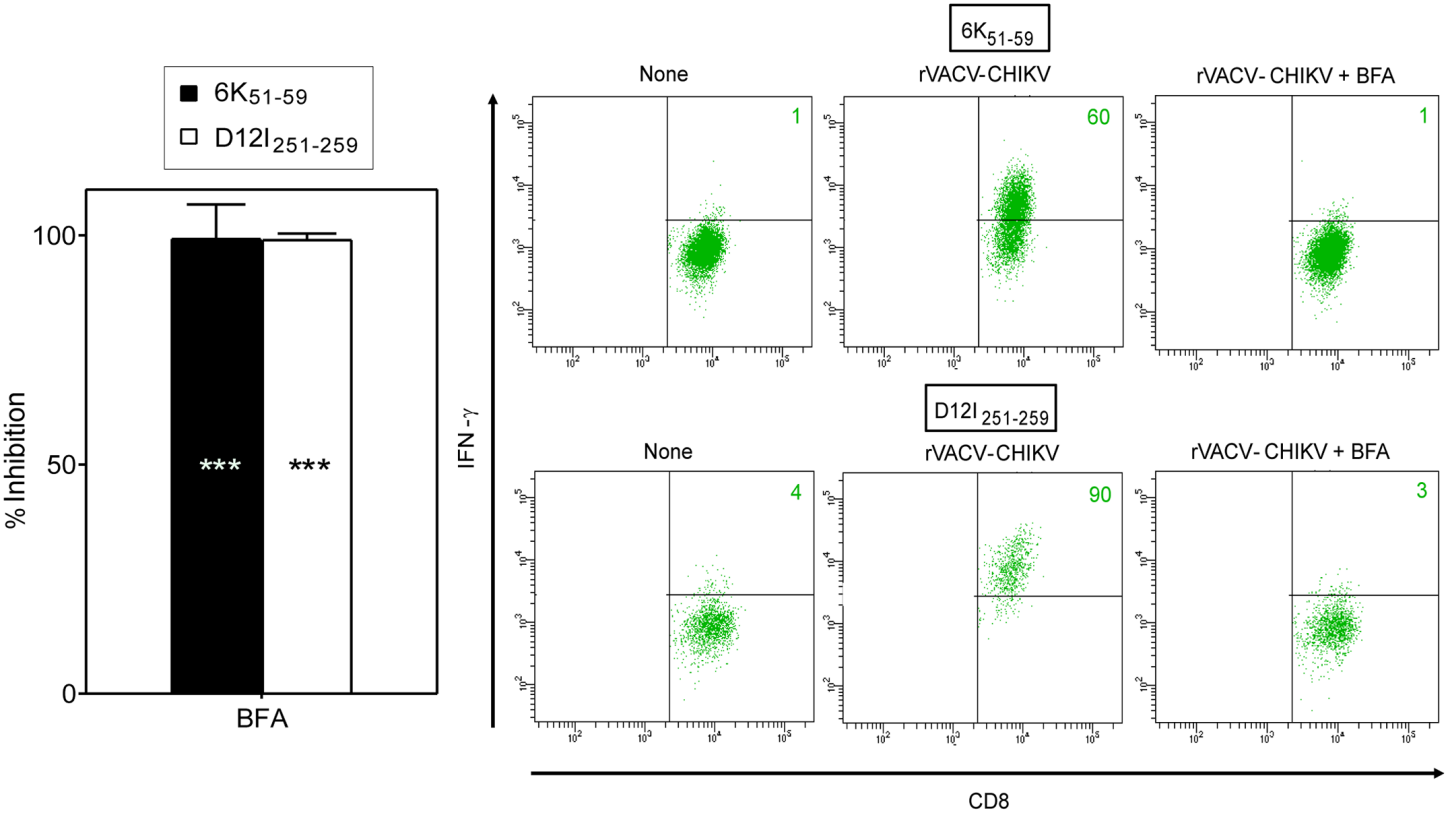
BFA effect on recognition of CHIKV 6K_51-59_ or VACV D12I_251-259_ viral epitopes. Mouse HLA-A*0201^+^ DC infected as in Fig 4 were treated with BFA. An ICS assay was used to test for recognition by CHIKV 6K_51-59_- or VACV D12I_251-259_-specific CD8^+^ T cell lines. Graph data are expressed as percentage of inhibition as by ICS in the presence of BFA (mean ± SD of four independent experiments; *** P <0.001 vs. respective rVACV-CHIKV-infected cells). Representative ICS panels with CHIKV 6K_51-59_- or VACV D12I_251-259_-specific CD8^+^ T cell lines are shown.

CHIKV 6K is a structural protein necessary both for virus budding and entry, which is incorporated in small amounts into the virion [11]. As rVACV-CHIKV expresses the five structural proteins of the pathogen, we cannot rule out the presence of CHIKV virus-like particles and possible exogenous antigen presentation. To test whether the CHIKV 6K_51-59_ HLA-A*0201-restricted epitope requires endogenous processing, we analyzed its presentation in the presence of BFA. Brefeldin A blocks class I export beyond the cis-Golgi compartment [31,32], preventing surface expression of newly assembled HLA class I-peptide complexes of endogenous origin (Table 1 summarizes the specificity of all inhibitors used). BFA addition during infection completely inhibited specific IFNγ secretion by the CHIKV 6K_51-59_ epitope-specific CD8^+^ T cell line (Fig 5), which demonstrated that this epitope was generated from CHIKV 6K protein endogenously processed in rVACV-CHIKV-infected cells. We also observed complete inhibition of specific IFNγ secretion by the VACV D12I_251-259_ epitope-specific CD8^+^ T cell line (Fig 5).

### A furin-like protease inhibitor specifically blocks CHIKV 6K_51-59_ epitope recognition

**Fig 6:**
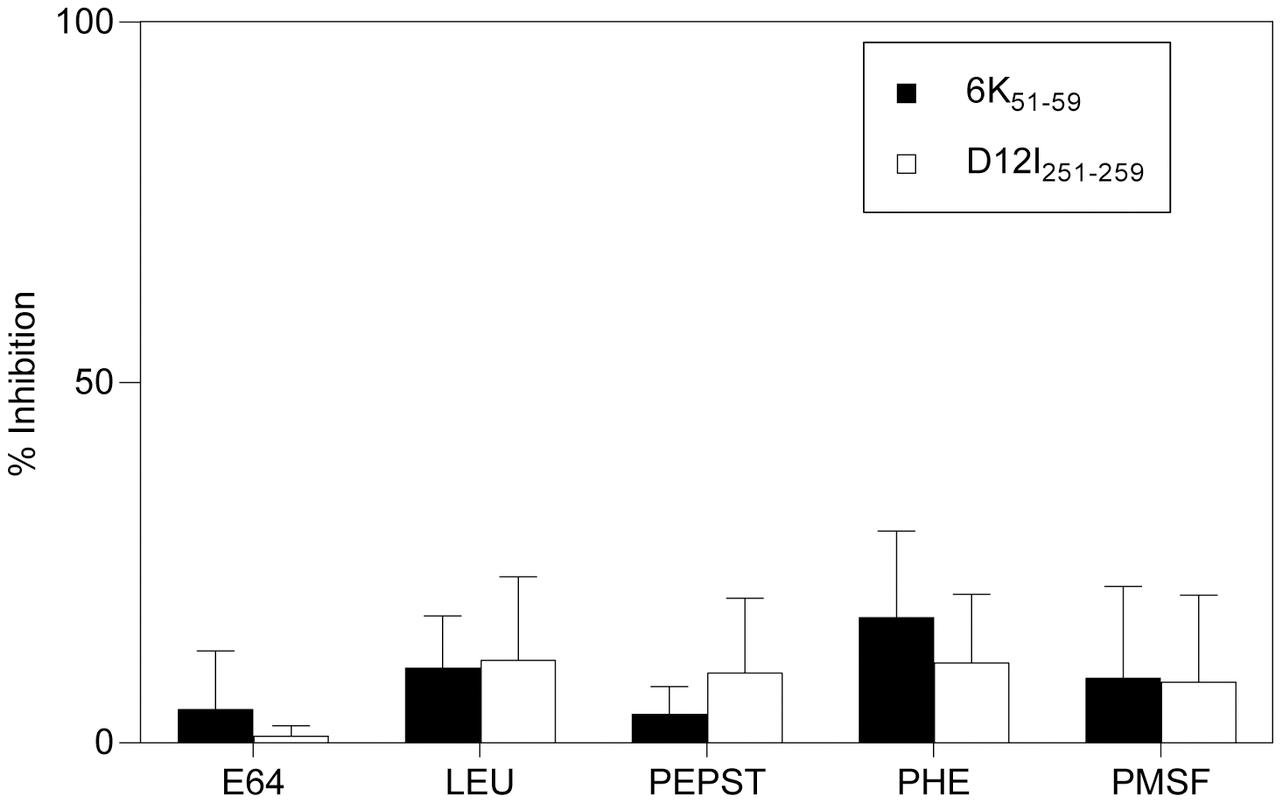
Recognition of infected HLA-A*0201^+^ DC by CHIKV 6K_51-59_- or VACV D12I_251-259_-specific CD8^+^ T cells in the presence of general peptidase inhibitors. Mouse HLA-A*0201^+^ DC infected as in Fig 4 were treated before ICS assay with the inhibitors E64 (cysteine protease C1), LEU (trypsin-like and cysteine protease), PEPST (aspartic protease), PHE (metallopeptidase), or PMSF (serine peptidases). CHIKV 6K_**51-59**_- or VACV D12I_251-259_-specific CD8^+^ T cell lines were used. The percentage of specific inhibition was calculated as in Fig 5. Data shown as mean ± SD of three independent experiments.

To study the antigen processing pathways involved in endogenous generation of the CHIKV 6K_51-59_ epitope, we performed ICS assays with several specific protease inhibitors on rVACV-CHIKV-infected cells. We tested E64 [33], leupeptin (LEU) [34], pepstatin (PEPST) [34,35], 1,10-phenanthroline (PHE), and phenylmethylsulfonyl fluoride (PMSF) [36] inhibitors, as they are specific for different protease families and cover a wide range of protease classes (Table 1). None of these inhibitors affected specific recognition of rVACV-CHIKV-infected target cells by the CHIKV 6K_51-59_-specific CD8^+^ T cell line (Fig 6). The enzymes inhibited by these drugs are thus not involved in generation of this epitope.

**Fig 7:**
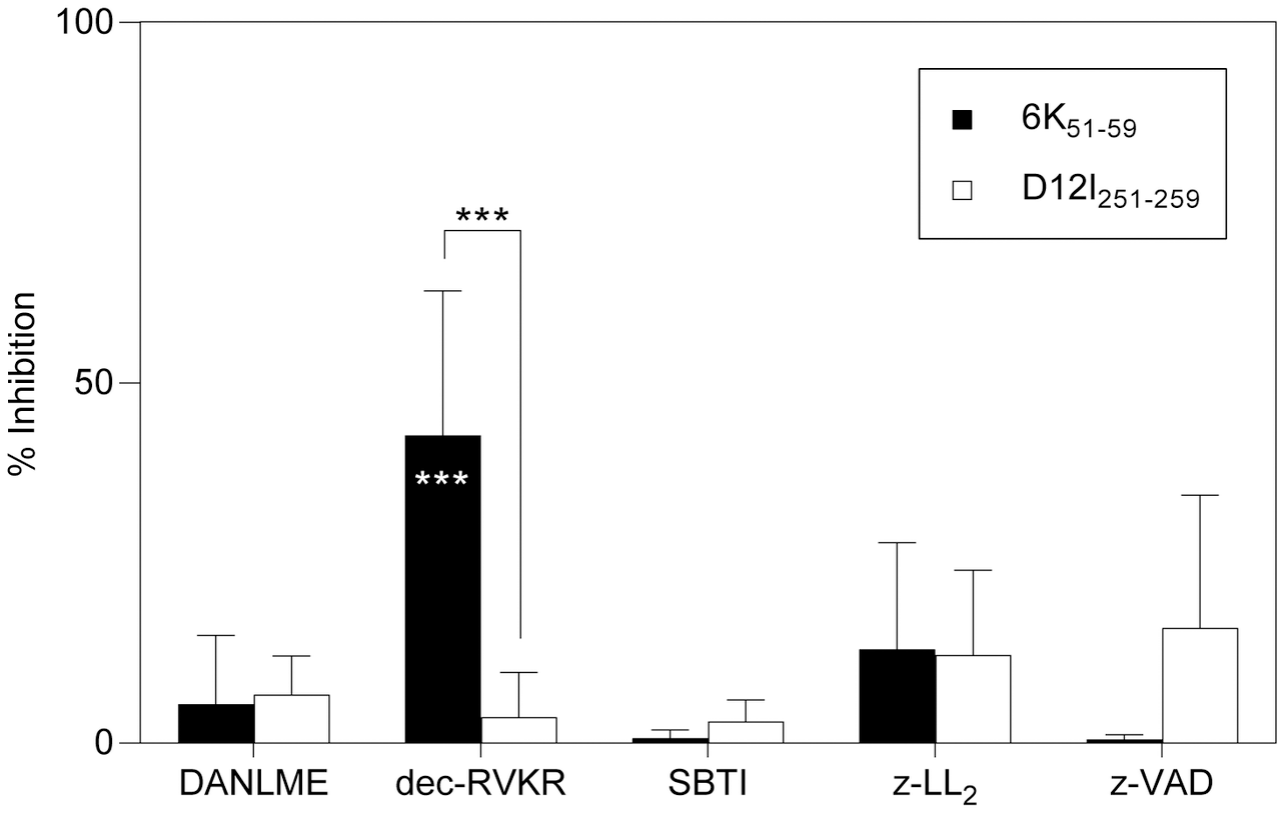
Recognition of infected HLA-A*0201^+^ DC by CHIKV 6K_51-59_- or VACV D12I_251-259_-specific CD8^+^ T cells in the presence of various protease inhibitors. Mouse HLA-A*0201^+^ DC infected as in Fig 4 were treated before ICS assay with protease inhibitors including DANLME (aspartic protease A1 inhibitor), dec-RVKR (furin and other members of the propotein convertase family), STBI (trypsin, chymotrypsin and plasmin), Z-LL_2_ (signal peptide peptidase), or zVAD (caspase). The percentage of specific inhibition was calculated as in Fig 5. Data shown as mean ± SD of four independent experiments (*** P <0.001 of CHIKV 6K_51-59_-specific CD8^+^ T cells vs. no inhibitor (white asterisks) or VACV D12I_251-259_ ligand with the inhibitor (black asterisks)).

We also tested specific inhibitors of several cellular proteases, most of which were not relevant for antigen processing of the CHIKV 6K_51-59_ viral epitope (Fig 7). In contrast, dec-RVKR, an inhibitor of furin and other proprotein convertases (Table 1), partially inhibited CHIKV 6K_51-59_-specific CD8^+^ T cell recognition of infected cells (43 ± 20%; Fig 7). To exclude the possibility that this inhibition was due to toxic effects on target cells or on VACV replication rather than to a specific protease block, we performed parallel experiments using the rVACV-CHIKV-infected target cells with another T cell line. These infected cells were recognized efficiently by the VACV D12I_251-259_-specific CD8^+^ T cell line, and no inhibition was detected (4 ± 6%; Fig 7). These data indicate that the dec-RVKR-induced inhibition of specific recognition by CHIKV 6K_51-59_-restricted CD8^+^ T cells was due to protease blockade and not to nonspecific effects. These data indicate that proprotein convertases are involved in the generation of the CHIKV 6K epitope.

### Dipeptidyl-peptidase II (DPPII) is involved in antigen processing and presentation of the CHIKV 6K_51-59_ epitope

**Fig 8:**
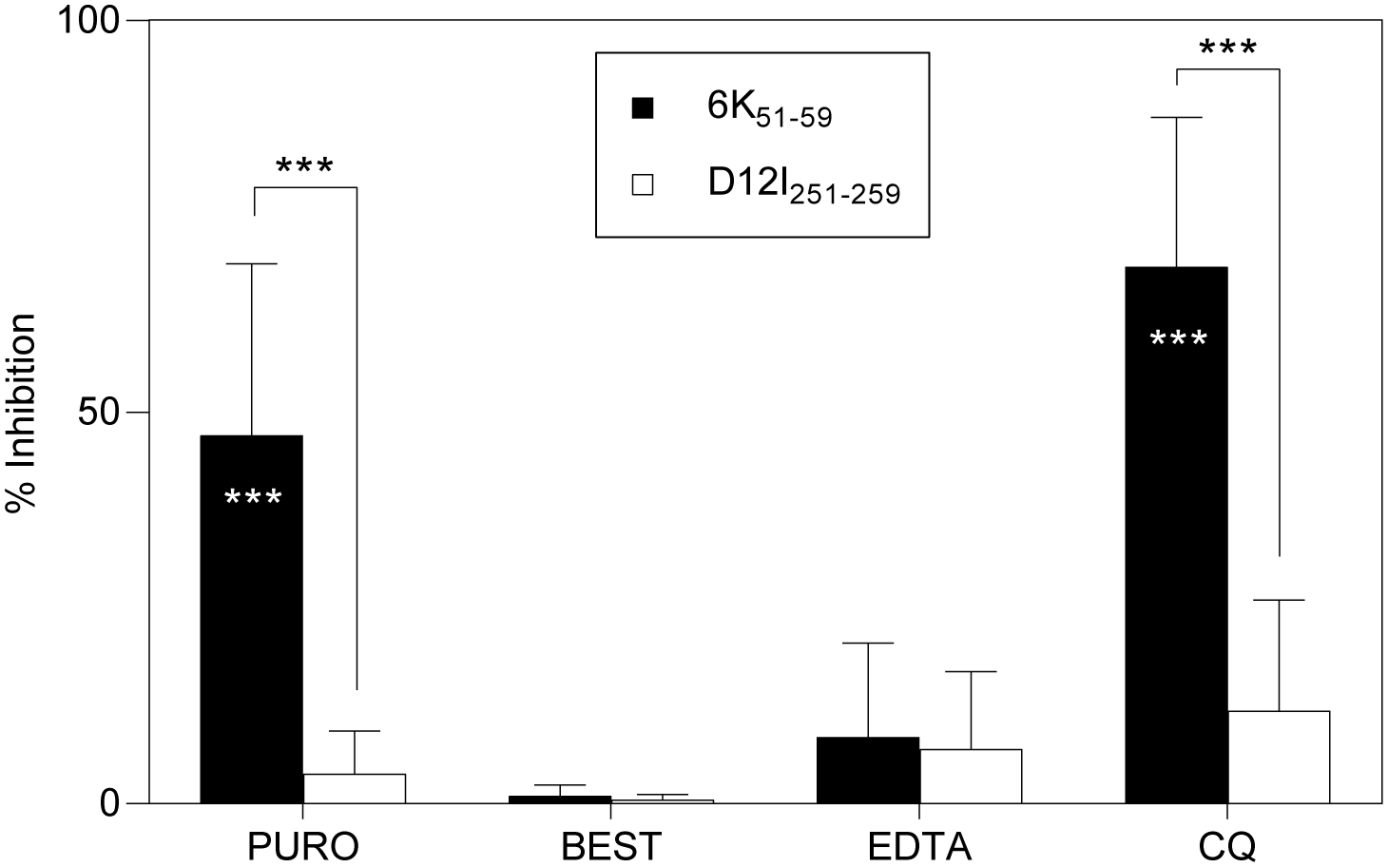
Effect of several inhibitors on recognition of rVACV-CHIKV-infected cells. Mouse HLA-A*0201^+^ DC infected as in Fig 4 were treated before ICS assay with inhibitors such as PURO (cytosol alanyl aminopeptidase and lysosomal DPPII inhibitor), BEST (metallo-aminopeptidases), EDTA (metallopeptidases and some cysteine proteases), or CQ (lysosomotropic agent). The percentage of specific inhibition was calculated as in Fig 5. Data shown as mean ± SD of four independent experiments (*** P <0.001, as in Fig 7).

The inhibitor puromycin (PURO) [37] (Table 1) partially blocked specific recognition of rVACV-CHIKV-infected target cells by CHIKV 6K_51-59_-specific CD8^+^ T cells (47 ± 21%), but had no effect on VACV D12I_251-259_ epitope presentation (4 ± 6%) (Fig 8). PURO is a reversible inhibitor of the cytosol alanyl aminopeptidase and of lysosomal DPPII. To identify the specific peptidase involved in CHIKV 6K_51-59_ peptide processing, we treated rVACV-CHIKV-infected target cells with additional inhibitors. CHIKV 6K_51-59_-specific CD8^+^ T cell recognition was unaffected by two distinct inhibitory compounds that block cytosol alanyl aminopeptidase activity, bestatin (BEST) and EDTA (ethylenediaminetetraacetic acid) (Table 1 and Fig 8), which excludes this cytosolic enzyme from antigen processing of the CHIKV 6K_51-59_ epitope. The antimalarial drug chloroquine (CQ), a lysosomotropic agent that affects DPPII and other lysosomal enzymes (Table 1), nonetheless blocked recognition of infected cells by CHIKV 6K_51-59_-specific CD8^+^ T cells (69 ± 19%; Fig 8). These data indicate that DPPII is involved in CHIKV 6K epitope generation.

### Sequential furin-like protease and DPPII activity are necessary for CHIKV 6K_51-59_ epitope generation

**Fig 9:**
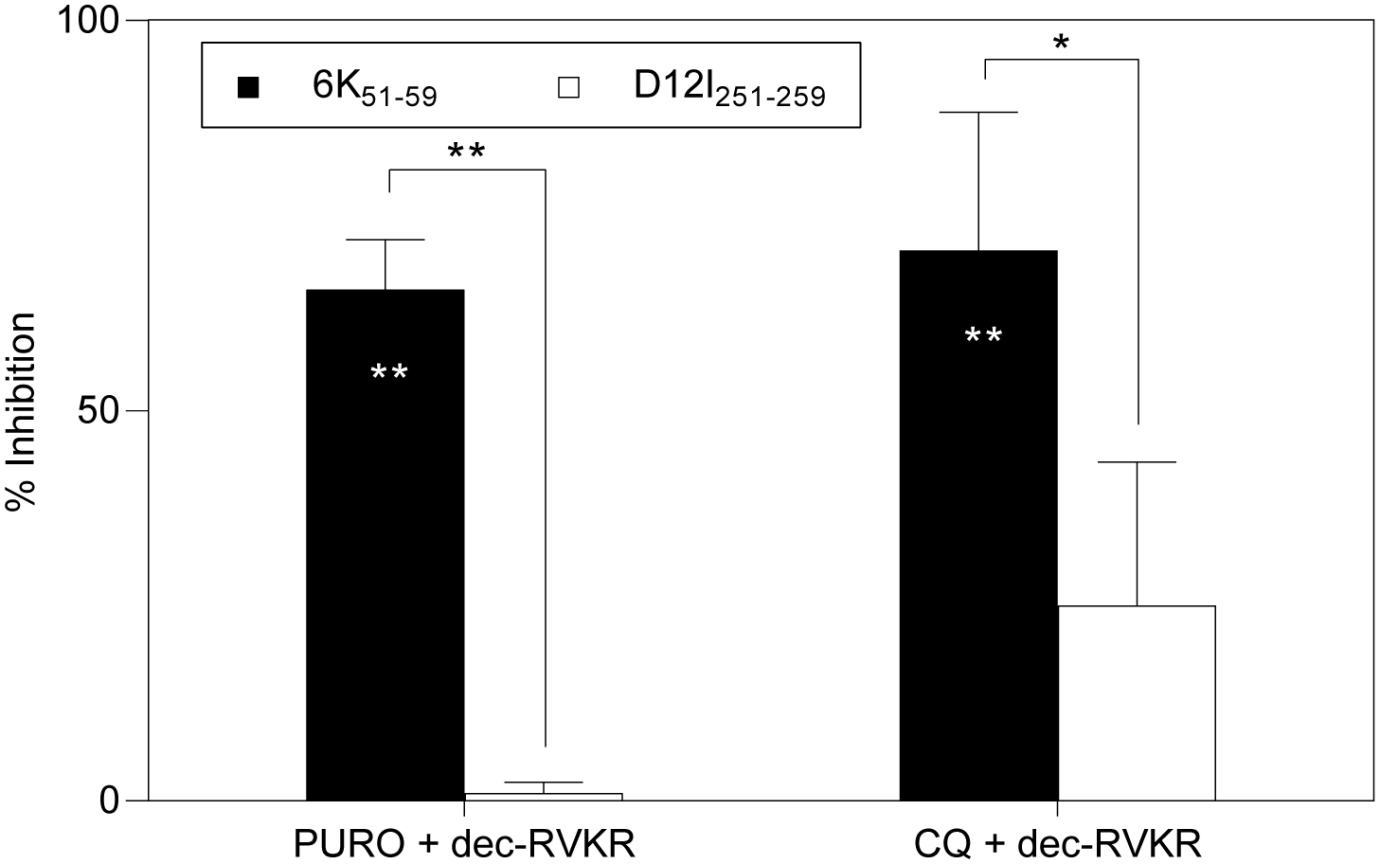
Effect of inhibitor combinations in recognition of rVACV-CHIKV-infected DC. Mouse HLA-A*0201^+^ DC infected as in Fig 4 were treated before ICS assay with a combination of dec-RVKR and CQ or PURO. The percentage of specific inhibition was calculated as in Fig 5. Data shown as mean ± SD of three independent experiments (** P <0.01; * P <0.05, as in Fig 7).

The inhibition of antigen recognition by dec-RVKR (Fig 7) or PURO (Fig 8) indicated that furin-like proteases and DPPII peptidase are both involved in antigen presentation of the CHIKV 6K_**51-59**_ epitope. The similar partial inhibition of rVACV-CHIKV-infected cell recognition by both drugs (dec-RVKR, 43 ± 20%; PURO, 47 ± 21%) is compatible with two explanations. The CHIKV 6K_**51-59**_ epitope might be processed sequentially by the two proteases. Alternatively, this epitope could be processed in parallel by proprotein convertases or by DPPII independently; in this case, both antigen processing pathways would have to be inhibited simultaneously to fully abrogate CHIKV 6K_**51-59**_ epitope presentation. To discriminate between these possibilities, we analyzed the effect on antigen presentation of the combined inhibitors on rVACV-CHIKV-infected cells. We observed a moderately increased blockage of presentation in target cells treated simultaneously with PURO and dec-RVKR (66 ± 6%; Fig 9), comparable and not statistically different to that observed when CQ and dec-RVKR were combined (71 ± 18%; Fig 9) or with CQ alone (69 ± 19%; Fig 8). The inhibitory effect of PURO and dec-RVKR was CHIKV 6K epitope-specific, as recognition of the VACV D12I_251-259_ epitope was not reduced in their presence (Fig 9). These results show that furin-like proteases and DPPII are found in the same CHIKV 6K_**51-59**_ epitope presentation pathway.

### Proteasome and ERAP are involved in antigen processing of the CHIKV 6K_51-59_ epitope

**Fig 10:**
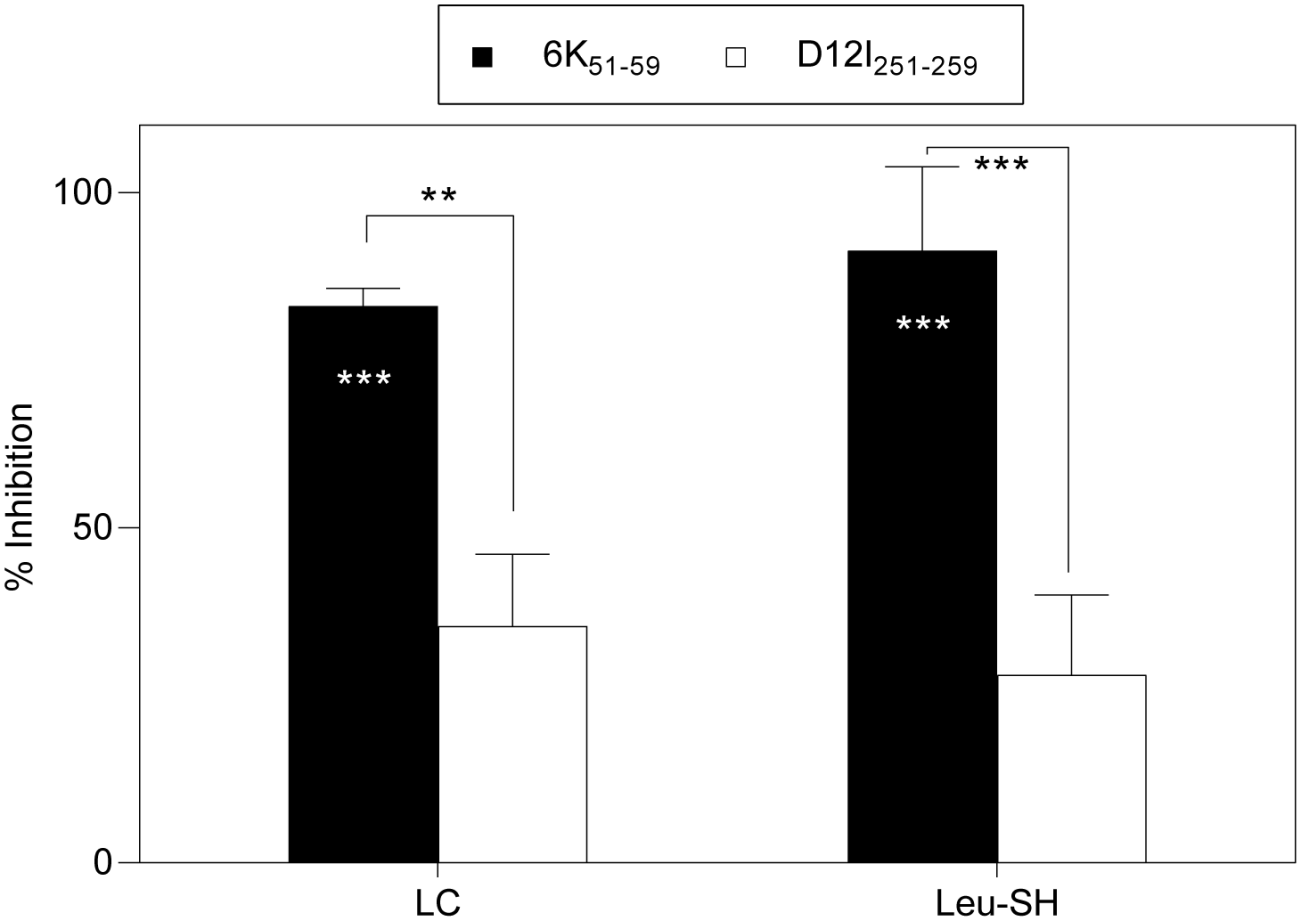
Effect of LC and Leu-SH inhibitors on recognition of CHIKV 6K_51-59_- or VACV D12I_251-259_ viral epitopes. Mouse HLA-A*0201^+^ DC infected as in Fig 4 were treated before ICS assay with LC (proteasome inhibitor) or Leu-SH (ERAP and other metallo-aminopeptidases). The percentage of specific inhibition was calculated as in Fig 5. Data shown as mean ± SD of four independent experiments (*** P <0.001; ** P <0.01, as in Fig 7).

To test whether the classical antigen processing pathway is involved in CHIKV 6K_51-59_ epitope generation, we used the proteasome inhibitor lactacystin (LC) [38,39], and leucinthiol (Leu-SH), which has activity against ERAP and other metallo-aminopeptidases [40] (Table 1). Both LC (83 ± 3%) and Leu-SH (91 ± 13%) blocked specific recognition of rVACV-CHIKV-infected target cells by CHIKV 6K_**51-59**_-specific CD8^+^ T cells (Fig 10). In contrast, in the same experiment, these drugs had a lesser effect on VACV D12I_251-259_ epitope presentation (Fig 10).

### Differential contribution of peptidases to antigen presentation of the CHIKV 6K_51-59_ epitope

CHIKV 6K_**51-59**_ epitope presentation to a specific T cell line was partially blocked by dec-RVKR (43 ± 20%), PURO (47 ± 21%) or both (66 ± 6%) (Figs 7, 8 and 9), whereas CHIKV 6K_**51-59**_ recognition by these CD8^+^ T cells was strongly inhibited by LC (83 ± 3%) and Leu-SH (91 ± 13%) (Fig 10). These differences were statistically significant (Table 3), which suggested that the CHIKV 6K_**51-59**_ epitope is generated by two distinct pathways, the classical antigen processing pathway and a second antigen presentation pathway that includes the four proteases (dec-RVKR, PURO, LC and Leu-SH).

**Table 3:** Statistical analysis of the inhibition values with various inhibitors.

	LC	Leu-SH	dec-RVKR	PURO	dec-RVKR + PURO
LC	-	ns ^a^	_**_ ^b^	_*_	_*_
Leu-SH		-	_***_	_***_	_*_
dec-RVKR			-	ns	ns
PURO				-	ns
dec-RVKR + PURO					-

^a^ Not significant

^b^ Significant P values: ***, p <0.001; **, p <0.01; *, p <0.05

## Discussion

In this study, we undertook identification of HLA-A*0201 epitopes from the CHIKV 6K protein and explored their antigen presentation pathways. Our results define several CHIKV 6K protein restricted epitopes, being to our knowledge the first time that epitopes from CHIKV are defined associated to human MHC class I molecules. Extended epitope prediction using the SYFPEITHI tool suggests that ligands of this small viral protein could be presented by a notable proportion of the HLA class I alleles tested (12 of 30; 40%, S1 Table). According to the Immune Epitope Database (IEDB) population coverage tool (http://tools.iedb.org/population/), these class I molecules are present in 86% of the human population (S2 Table). The short viral CHIKV 6K protein is thus of interest for targeting the cellular immune system. Further studies are needed to analyze cellular immune responses in CHIKV-infected individuals.

**Fig 11:**
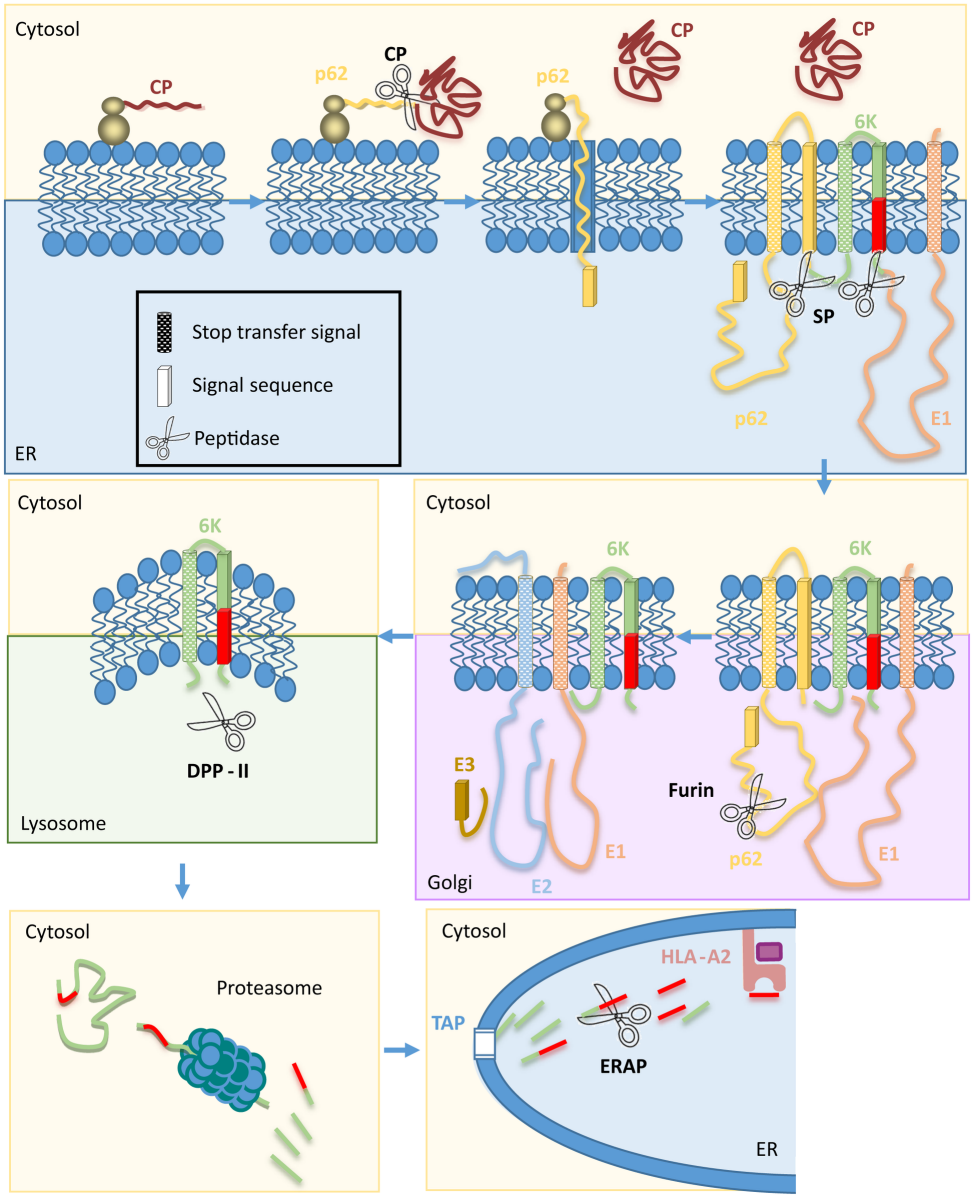
Diversity of proteases and processing pathway involved in CHIKV 6K_51-59_ epitope presentation. The model shows the components of the antigen presentation pathway proposed for the CHIKV 6K_51-59_ epitope. Stop-transfer signals are indicated by rectangular blocks, and signal sequences by dashed cylinders. Subcellular organelles are shown as colored boxes: cytosol (yellow), ER (blue), trans-Golgi network (mauve) and lysosomes (green). CHIKV proteins are capsid (CP, maroon), p62 (yellow), 6K (green), E1 (peach), E2 (blue), and E3 (brown). The CHIKV 6K_51-59_ epitope is depicted in red. The role of the distinct proteases is deduced from CD8^+^ T cell sensitivity to the various inhibitors, except for the signal peptidase, whose role was described in the generation of the Alphavirus 6K protein [59].

Here we identified three HLA-A*0201-restricted epitopes in the CHIKV small 6K protein. Using several protease inhibitors (Table 1), we report that various proteolytic activities (probably in two distinct antigen processing pathways) are necessary to generate one of these epitopes, the CHIKV 6K_51-59_ epitope. These results are consistent with a model for CHIKV maturation and processing and, by extrapolation, that of other *Alphavirus* structural polyproteins (Fig 11). Although no furin cleavage motif was found in the 6K protein, 6K_51-59_ peptide presentation was dependent on dec-RVKR-sensitive proteases, which indicates that proprotein convertase activity is needed to generate this epitope. Like other host and viral proteases [41], furin are involved in processing structural polyproteins in all Alphaviruses to yield the mature structural proteins that will form the virion. Maturation of the CHIKV structural polyprotein thus affects antigen processing of the 6K_51-59_ epitope.

With regard to the CHIKV replication cycle, only limited information can be extrapolated from comparison between CHIKV structural proteins and those of other *Alphaviruses*. The translation order of these Alphavirus polyproteins is capsid, PE2 precursor (that includes envelope glycoproteins E3 and E2, 6K, and envelope protein E1) [41]. Immediately after the ribosome starts translation of the PE2 precursor, the capsid protein (whose C-terminal domain has protease activity) is released in the cytosol by autoproteolysis. The new N terminus of the polyprotein thus bears a signal sequence for translocation of the PE2 precursor across the ER membrane. Additional signal sequences in the C terminus of E2 and 6K proteins allow their translocation to the ER. In the ER, signal peptidase cleavage of the C terminus of both the PE2 precursor and the 6K protein releases three viral protein products (PE2, 6K, and E1). The PE2 precursor and E1 protein remain attached to the membrane by their C terminus, and 6K remains as a short transmembrane protein. In the Alphavirus Sindbis virus, E1 and PE2 glycoproteins form a heterodimer in the ER, and this interaction is sufficient for transport beyond this organelle [42]. The E1-PE2 heterodimer reaches the trans-Golgi network, but prior to the cell membrane, CHIKV PE2 is cleaved by furin and other proprotein convertases such as PC5A, PC5B, and PACE4 to generate the mature E2 and E3 proteins [43]. CHIKV 6K_51-59_ epitope-dependent presentation by dec-RVKR-sensitive proteases thus indicates that CHIKV envelope proteins are transported from the ER to the trans-Golgi network as heterotrimers that also include CHIKV 6K protein, as also described for Semliki Forest virus [44]. In Alphaviruses, this cleavage induces conformational changes in E1 and E2 proteins, thus promoting extensive contacts between these two proteins to yield the spike architecture of activated viral envelope complex in 1:1 stoichiometry [41]. The role of the E3 structural protein is unclear; E3 is associated with virions in Semliki Forest virus [45], but not in other Alphaviruses including CHIKV [46, 47].

In Semliki Forest and Sindbis viruses, substoichiometric amounts of 6K are incorporated into the virion [44,48]. Most of this small protein must thus be discarded in the infected cells, although the fate of the CHIKV 6K protein is nonetheless unclear. As both PURO and CQ impaired antigen recognition of target cells by CHIKV 6K_51-59_-specific CD8^+^ T cells, the lysosomal DPPII must have a role in processing this epitope. This data also indicated that at least a fraction of the CHIKV 6K protein must be degraded in the lysosomes.

DPPII-processed CHIKV 6K protein or a fragment that includes the viral epitope must be transported to the cytosol for proteasome processing, as indicated by LC inhibition of the CHIKV 6K_51-59_ antigen presentation. How these fragments reached the cytosol remains unclear, but the proteasome is involved in the generation of some epitopes of the Epstein-Barr virus (EBV) latent membrane protein 2 (LMP2) transmembrane nucleoprotein, albeit by uncharacterized mechanisms [49]. Host cell transmembrane protein processing might be involved in both CHIKV and EBV epitopes [50].

The block in CHIKV 6K_51-59_-specific recognition by Leu-SH, but not by two drugs that do not inhibit ERAP activity (the general metalloproteinase inhibitor PHE and the aminoprotease inhibitor BEST) [51,52], indicates that ERAP or a similar metalloproteinase produces the final CHIKV 6K_51-59_ epitope, probably in the ER, after transport of proteasomal products by TAP.

The statistically different percentages of inhibition by LC and Leu-SH inhibitors vs. dec-RVKR, PURO and CQ drugs also suggest a direct contribution of the classical antigen processing pathway, with proteasome degradation of DRiPs from viral polyprotein followed by ERAP trimming. The relative contribution of both pathways to antigen presentation was quantified using the same percentage of inhibition from one-third to half by the classical antigen processing pathway and half to two-thirds by the circular antigen presentation pathway. Our results show a broad diversity of proteases involved in a complex antigen presentation pathway to yield the viral CHIKV 6K epitope.

In addition to proteasome and ERAP, several proteases are implicated in processing endogenously synthesized HLA class I antigens (reviewed in [19]). Many proteases included here, such as signal peptidase [53,54], furin [55,56], and uncharacterized lysosomal CQ-sensitive enzymes [57,58], have been linked independently to the processing of several epitopes, although sequential activity of these peptidases to generate a specific HLA class I epitope has not been described. These proteases and the supplementary involvement of DPPII in CHIKV 6K_51-59_ antigen presentation define the most complex antigen processing and presentation pathway reported to date; this route begins in the ER and includes the trans-Golgi network, lysosomes, retrograde transport to cytosol, and ER re-entry.

Lastly, the results reported here also have implications for analysis of the cellular immune response. Only proteasome and ERAP, but not other protease inhibitors, are generally used to analyze antigen presentation of different HLA class I ligands or epitopes. Inhibition is normally sufficient to formally assign presentation of an epitope to the classical antigen processing pathway, excluding additional protease activities (Fig 10). In addition to the *Alphavirus* genus and the *Togaviridae* family, however, many viruses of different families and orders use host proteases from distinct subcellular locations to generate mature envelope and even nuclear proteins. In other viral epitopes, it would thus not be unexpected to find complex antigenic processing and presentation pathways similar to those reported here, if the antiviral cellular immune response was analyzed in depth with a broad spectrum of protease inhibitors as was carry out in the current investigation.

In conclusion, the results of the present report highlight the diversity of peptidases involved in HLA class I antigen presentation and expose the complexity of antigen processing pathways, as represented by the CHIKV 6K protein. Definition of the importance of this epitope in natural infection nonetheless awaits studies in CHIKV-infected individuals. This process could have broad implications when applied to other viral proteins.

## Supporting information

S1 Table Potential candidates to HLA-A or -B epitopes from CHIKV 6K protein. (DOCX)

S2 Table HLA class I coverage in different populations. (DOC)
