# Supplementary material for: Complex antigen presentation pathway for an HLA-A*0201-restricted epitope from Chikungunya 6K protein
Source: PLoS Negl Trop Dis. 2017 Oct 30;11(10):e0006036. doi: 10.1371/journal.pntd.0006036 (PMC5679651; doi:10.1371/journal.pntd.0006036)
Supplement: S1 Table — (DOCX) [file pntd.0006036.s001.docx]

Table S1

# Potential candidates to HLA-A or -B epitopes from CHIKV 6K protein

| Allele | 9 mers ^a^ | 10 mers ^b^ | Total |
| --- | --- | --- | --- |
|  |  |  |  |
| HLA-A*01 | 1 | 0 | 1 |
| HLA-A*02 | 5 | 3 | 8 |
| HLA-A*03 | 1 | 2 | 3 |
| HLA-A*11 | 0 | 0 | 0 |
| HLA-A*24 | 1 | 0 | 1 |
| HLA-A*26 | 0 | 1 | 1 |
| HLA-A*68 | 0 | 0 | 0 |
| HLA-B*07 | 1 | 0 | 1 |
| HLA-B*08 | 1 | 0 | 1 |
| HLA-B*13 | 1 | 0 | 1 |
| HLA-B*14 | 0 | 0 | 0 |
| HLA-B*15 | 0 | 0 | 0 |
| HLA-B*18 | 0 | 0 | 0 |
| HLA-B*27 | 1 | 0 | 1 |
| HLA-B*35 | 1 | 0 | 1 |
| HLA-B*37 | 0 | 0 | 0 |
| HLA-B*38 | 0 | 0 | 0 |
| HLA-B*39 | 0 | 0 | 0 |
| HLA-B*40 | 0 | 0 | 0 |
| HLA-B*41 | 0 | 0 | 0 |
| HLA-B*42 | 0 | 0 | 0 |
| HLA-B*44 | 0 | 0 | 0 |
| HLA-B*45 | 0 | 0 | 0 |
| HLA-B*47 | 0 | 0 | 0 |
| HLA-B*49 | 0 | 0 | 0 |
| HLA-B*50 | 0 | 0 | 0 |
| HLA-B*51 | 1 | 0 | 1 |
| HLA-B*53 | 0 | 0 | 0 |
| HLA-B*57 | 0 | 0 | 0 |
| HLA-B*58 | 1 | 0 | 1 |

^a^ and ^b^: number of nonamers and decamers with score > 20 from epitope prediction tool SYFPEITHI (<http://www.syfpeithi.de>), respectively.
