# Supplementary material for: Complex antigen presentation pathway for an HLA-A*0201-restricted epitope from Chikungunya 6K protein
Source: PLoS Negl Trop Dis. 2017 Oct 30;11(10):e0006036. doi: 10.1371/journal.pntd.0006036 (PMC5679651; doi:10.1371/journal.pntd.0006036)
Supplement: S2 Table — (DOC) [file pntd.0006036.s002.doc]

Table S2

# HLA class I coverage in different populations

| Area/Population | % Coverage^a^ |
| --- | --- |
|  |  |
| Amerindian | 75 |
| Arab | 78 |
| Asian | 72 |
| Black | 65 |
| Caucasoid | 96 |
| Hispanic | 85 |
| Jew | 78 |
| Kurd | 81 |
| Oriental | 71 |
| Polynesian | 83 |
| Siberian | 89 |
|  |  |
| World | 86 |
|  |  |

^a^ % coverage of 12 positive HLA class I alleles from Table S1 according to the Immune Epitope Database (IEDB) coverage population web tool (<http://tools.iedb.org/population/>)
